# Influence of Talc on the Properties of Silicone Pressure-Sensitive Adhesives

**DOI:** 10.3390/ma17030708

**Published:** 2024-02-01

**Authors:** Adrian Krzysztof Antosik, Artur Grajczyk, Marzena Półka, Magdalena Zdanowicz, John Halpin, Marcin Bartkowiak

**Affiliations:** 1 Department of Chemical Organic Technology and Polymeric Materials, Faculty of Chemical Technology and Engineering, West Pomeranian University of Technology in Szczecin, 70-322 Szczecin, Poland; marcin.bartkowiak@zut.edu.pl; 2 Faculty of Safety and Civil Protection Engineering, The Main School of Fire Service, Słowackiego 52/54, 01-629 Warsaw, Poland; grajczykartur@gmail.com (A.G.); mpolka@apoz.edu.pl (M.P.); 3 Center of Bioimmobilisation and Innovative Packaging Materials, Faculty of Food Sciences and Fisheries, West Pomeranian University of Technology in Szczecin, Janickiego 35, 71-270 Szczecin, Poland; magdalena.zdanowicz@zut.edu.pl; 4 Taconic, Forest Park, N91 E1WD Mullingar, County Westmeath, Ireland; johnhal@4taconic.com

**Keywords:** adhesion, PSA, silicone pressure-sensitive adhesives, self-adhesives tapes, talc

## Abstract

The article describes new silicone self-adhesive adhesives modified with the addition of talc. The obtained self-adhesive materials were characterized to determine their adhesive properties (adhesion, cohesion, and adhesion) and functional properties (pot life of the composition, shrinkage, and thermal properties of adhesives). Novel materials exhibited high thermal resistance above 225 °C while maintaining or slightly reducing other values (adhesion, cohesion, shrinkage, and tack). Selected composition: T 0.1 was used to prepare self-adhesives in industrial-scale production. Moreover, conducted test results revealed that the addition of talc delayed the thermal decomposition of the adhesive and provided reduced intensity of smoke emissions during combustion as well as the flammability of the adhesive layer.

## 1. Introduction

Silicone adhesives produced using multimolecular polymers are characterized by excellent physical properties. They are particularly characterized by the possibility of use in a wide temperature range, stable adhesive parameters, high aging resistance, and peel strength. Among them are silicone pressure-sensitive adhesives (Si-PSA), which maintain their adhesive properties at room temperature [1,2,3]. 

Si-PSA are usually composed of silane-functional polymers and silicone resins. Compared to other pressure-sensitive adhesive organic polymer adhesives, Si-PSA have much better properties. They are characterized by high chemical resistance, high flexibility of connections, and the possibility of use in an environment exposed to changing weather conditions. Their very important feature is also their ability to adhere to low-surface-energy materials. Moreover, they have favorable electrical parameters, such as perfect insulating properties, even at increased temperatures. They have no negative impact on the human body; hence, they are used even in the medical industry [4,5]. During cross-linking of silicone pressure-sensitive adhesives, the cohesion increases, and the stickiness and adhesion decrease at the same time. The type and amount of cross-linking agent used, as well as the cross-linking method, influence the mechanical and physicochemical properties. Platinum complexes or organic peroxides are standardly used to cross-link silicone self-adhesive adhesives [2,4,5,6,7,8].

Talc is a magnesium silicate mineral with the formula Mg_3_Si_4_O_10_(OH)_2_. It is a main constituent of soapstone rock, with a plate-like structure (lamellarity). Talc has a specific surface area of 3–35 m^2^/g, a density of 2.8 g/cm^3^, and a particle size of 1.4–19 μm. The layers in its lamellar structure are joined only by van der Waals force, so the shear strength is very low. The lamellar structure of talc results in special properties of talc-filled polymers, for example, low resistivity, low gas permeability, and increased thermal resistance. Talc has a hydrophobic character, so it is used as a filler in the majority of polymers [2,9], and its hydrophobicity can be additionally increased by coating it with zinc stearate. 

Talc is commonly used as a mineral filler for polymers, especially in thermoplastics for example, polypropylene, where talc helps to keep an appropriate balance between strength and stiffness and increases heat distortion temperature [9]. In this role, it is definitely better than cheaper calcium carbonates with a blocky structure, thanks to its lamellar structure (a higher aspect ratio as a ratio of length to diameter of the particle). Talc filler also improves the creep resistance of thermoplastics. Its use as a flame retardant in many plastics has also been described due to its thermophysical properties. [10,11,12,13,14,15].

Talc, next to clay, is the most commonly used filler for, but not limited to, rubber-based pressure-sensitive adhesives [2,16]. Unfortunately, there are no studies in the literature on its applications in silicone PSAs. This is a big loss because its characteristics, together with those of silicone PSA, indicate that a unique combination of thermal stability and durability of adhesive joints can be achieved.

The research aimed to fill this gap and develop new silicone pressure-sensitive adhesives modified with talc. The influence of the filler content on the properties of the obtained Si-PSA was determined using the SAFT test, thermogravimetric analysis (TGA), measurements of smoke release, and heat of combustion. The self-adhesive materials thus obtained with increased thermal resistance and fire safety were also obtained on an industrial scale to check the scalability of the technology for obtaining new Si-PSA.

## 2. Materials and Methods

### 2.1. Materials 

In the research, the following materials were used:

- Silicone resin DOWSIL™ 7358 (Q2-7358) from Dow Corning (Midland, MI, USA).
- NOVIPER DB 50—Bis(2,4-dichlorobenzoyl) peroxide (DClBPO) from Novichem (Chorzów, Poland)—cross-linking agent.
- Toluene from Carl Roth (Karlsruhe, Germany)—solvent.
- Talc from Elementis UK Ltd. (Cologne, Germany)—filler.

### 2.2. Pot Life

Pot life refers to the time when the viscosity of silicone resin increases (twice to four times when compared to the viscosity of the initial composition), making it impossible to apply the adhesive to the carrier film. The measurement was performed immediately after mixing the composition and was carried out at room temperature. Measurements of viscosity were carried out using the rotational viscometer IKA Rotavisc me-vi (IKA-Werke GmbH & Co. KG, Staufen im Breisgau, Germany).

### 2.3. Preparation of One-Sided Self-Adhesive Tape

#### 2.3.1. Laboratory Scale

**Figure 1:**
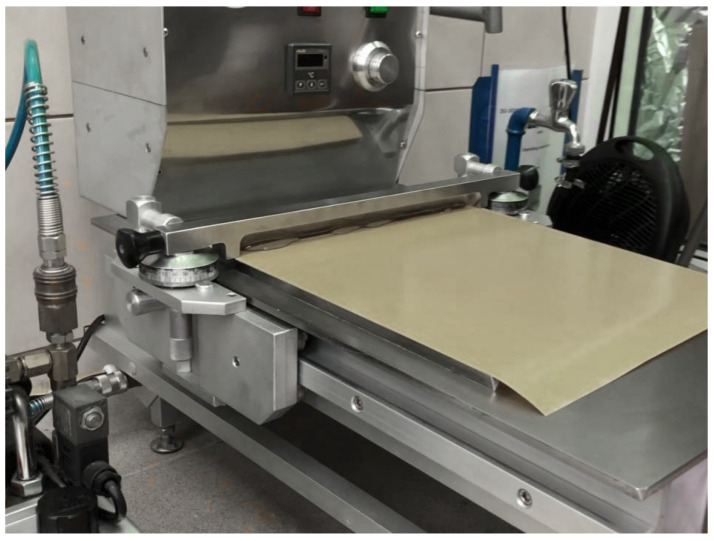
The laboratory coating machine used in the studies.

The silicone resin was mixed with toluene and the cross-linking agent until homogeneity (50 wt.% polymer; 1.5 wt.% 2,4-dichlorobenzoyl peroxide (DClBPO) based on polymer dry weight). Subsequently, talc powder was added in the following amounts: 0.1, 0.5, 1.0, and 3.0 pph (parts per 100 part of resin) and mixed. The sample acronyms are presented in Table 1. A polyester (PET) film with a thickness of 50 µm was coated with silicone composition at a speed of 5 cm/s to obtain an adhesive film and dried for 10 min at 120 °C in a drying channel. Such an adhesive film was protected with a fluorosilicone coated PET film (Dolpap Ltd., Chojnów, Poland). The single-side adhesive tapes obtained were used for adhesive tests. The laboratory coating machine is presented in Figure 1.

#### 2.3.2. Industrial Scale

The silicone resin was mixed with toluene and the cross-linking agent until homogeneity (50 wt. % polymer; 1.5 pph 2,4-dichlorobenzoyl peroxide (DClBPO) based on polymer dry weight). Subsequently, the wt. 0.1% amount of talc was added, calculated based on the dry weight of the polymer, and mixed. A one-side fluorosilicone coated film with a thickness of 50 µm was coated (on the non-fluorosiliconized side) by the silicone composition at a speed of 7 cm/s and dried at 100, 150, and 190 °C in subsequential drying channel zones to obtain an adhesive film. The adhesive film was rolled into a roll. Then, the resulting beam of adhesive tape was cut to obtain rolls of adhesive tape of the appropriate width. The single-side adhesive tapes obtained were used for adhesive tests.

### 2.4. Peel Adhesion

**Figure 2:**
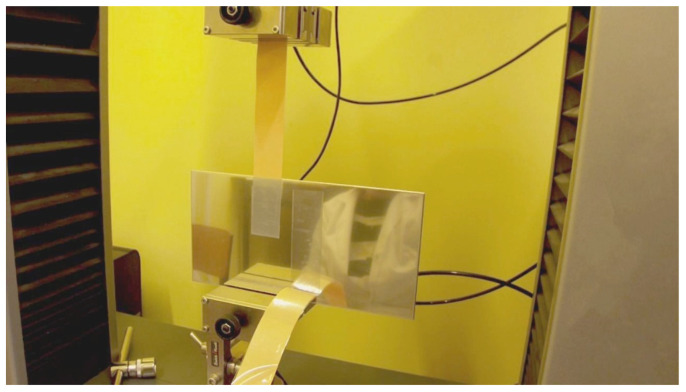
Peel adhesion test according to the FTM-1 standard.

Adhesion is defined as the interaction between the surfaces. It closely relates to interfacial attraction. The tests of adhesion were performed according to the industrial standard FTM 1 [17], entitled Peel Adhesion (180°) at 300 mm per minute, by FINAT (Fédération Internationale des Fabricants et Transformateurs d’Adhesifs et Thermocollants sur Papiers et Autres Support). Peel adhesion is the force required to peel off a PSA-coated flexible material from the standard surface at a specified angle and speed. According to the FTM-1 standard, the angle was 180°, and the speed of removal was 300 mm/min. The method was followed according to the standard. A strip of PSA-coated material (25 × 175 mm) was partially applied to the steel plate using a 2 kg standard roller. After 20 min of contact time, the sample was ready to continue the test. The free end of the strip and the plate were mounted in the grips of the tensile testing machine, so the angle of the peel was 180°, as shown in Figure 2. The grip separation rate was set at 300 mm per minute. The average value of the force was calculated from a few measurements taken from the middle area of the test strip. The temperature of the test was set to 23 °C.

### 2.5. Cohesion

**Figure 3:**
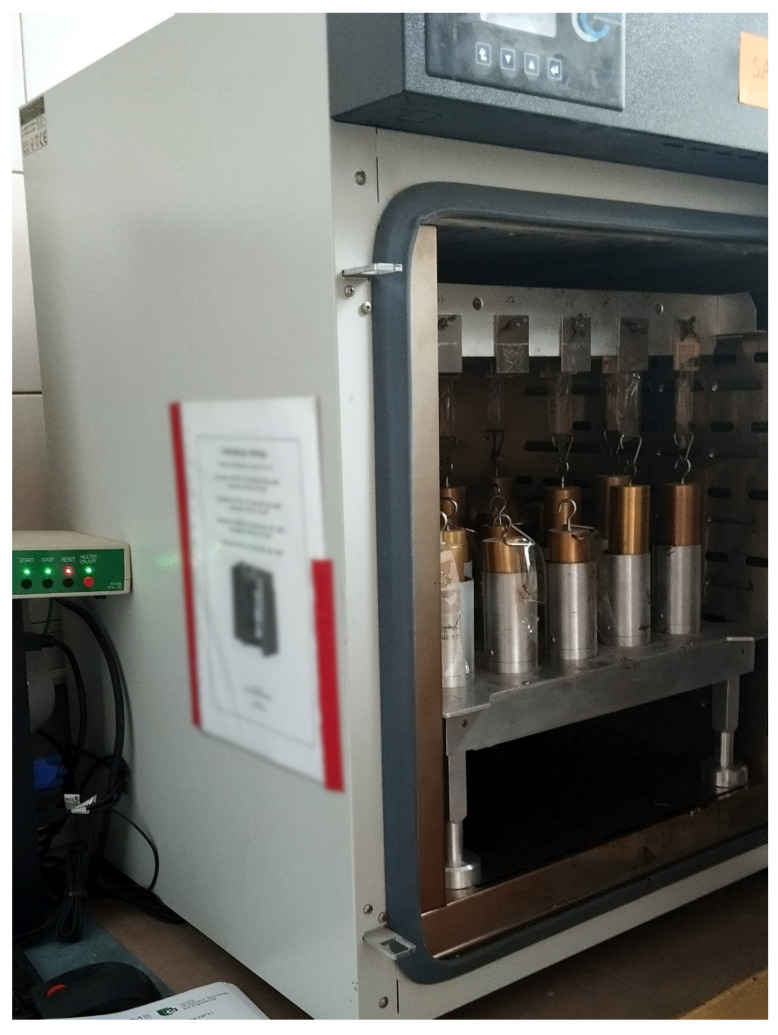
Cohesion test according to the FTM-8 standard.

Cohesion refers to the internal strength of the pressure-sensitive adhesive joint. It depends mainly on temperature, the cross-linking agent content, and the thickness of the adhesive film. Shear strength was determined due to the standard FINAT FTM 8 [17], entitled Resistance to Shear from a Standard Surface. This method measures the ability of the PSA layer to withstand static forces applied parallelly to the sample. The measurements were conducted at room temperature (23 °C) and, optionally, at an elevated temperature (70 °C). The method according to the standard was followed. A strip of PSA-coated material (25 × 175 mm) was applied to the test steel plate on a length of 25 mm, so the contact area between substrate and adhesive was 25 × 25 mm. The steel plate with the sample strip was held in the rack, and the free end of the strip was loaded with 1kg of hanging weight. The rack with the desired number of samples was hung in the chamber at a stabilized temperature, as shown in Figure 3. The time elapsed for each sample to separate from the test plate was measured by an automatic sensor. 

### 2.6. Tack

**Figure 4:**
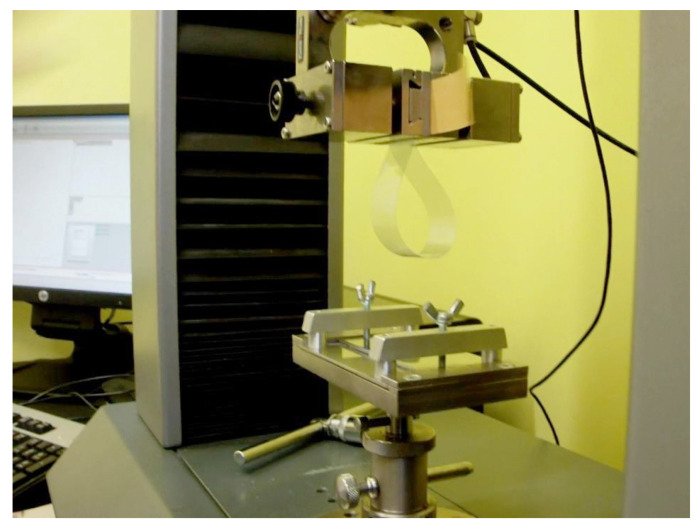
Loop tack test according to the FTM-9 standard.

The tack is a very important property of pressure-sensitive adhesives, sometimes called “initial grab”. Tack refers to the ability of an adhesive to bond at low pressure with another surface. It can also be defined as the force needed to separate the surfaces after a short contact time. It was determined using the technical standard FINAT FTM 9 [17], entitled Loop Tack Measurement. The standard requires a tensile testing machine and a properly prepared sample, as presented in Figure 4. A strip of PSA-coated material with dimensions of 25 mm width and a minimum of 175 mm length was folded to form the loop with the adhesive facing outermost and then mounted in the upper grips of the tensile tester. A flat plate made of glass or steel with dimensions of 25 × 30 mm was mounted horizontally in the lower grips of the tester. Next, the machine was started to bring the loop into contact with the plate until the testing area of 25 × 25 mm was covered with the sample. Then, immediately, the direction of the grip movement was reversed, and the separation started at a rate of 300 mm per minute. Then, the force was measured by the machine.

### 2.7. Thermal Resistance

To determine the thermal resistance, the SAFT (shear adhesion failure temperature) test was performed. The measurement system is constructed similarly to cohesion measurements (2.5). However, the test was not performed at a constant temperature, but a programmed temperature increase was performed. The samples were prepared according to the procedure described for the cohesion tests. The temperature was increased from 23 to 225 °C at a heating rate of 1 °C/min. As a result, the damage temperature was given along with the nature of the adhesive damage. Tests were carried out on 4 samples for each formulation to determine the mean thermal resistance.

### 2.8. Shrinkage

The shrinkage test was carried out according to the industrial standard FINAT FTM 14 [17], entitled Dimensional Stability. This method was as follows: the PVC foil (10 cm × 10 cm) with adhesive film was applied onto an aluminum plate using the roller and conditioned at 23 °C for 72 h. After conditioning, the sample was trimmed to the dimension of the plate, and in the center of the film, vertical and horizontal incisions were made to form a cross (incisions with a dimension of 8 cm). Subsequently, the sample was seasoned at 70 °C for 7 days in the oven. The shrinkage was determined after 10 and 30 min; 1, 3, 8, and 24 h; and after 2, 3, 4, 5, 6, and 7 days (width of the slits formed by the cuts).

### 2.9. Differential Scanning Calorimetry

The cross-linking process of the resin composition has a measurable thermal effect (exothermic peak), which can be tested using differential scanning calorimetry. DSC Apparatus Q100 by TA Instruments (New Castle, DE, USA) was used during the research.

The measurements were carried out under a nitrogen atmosphere, and the samples were scanned from −100 to 300 °C at a heating rate of 5 °C/min.

### 2.10. Thermogravimetric Analysis 

The thermogravimetric analysis of adhesive composition was carried out using the TGA 500Q thermogravimeter from TA Instruments (New Castle, DE, USA). The test was carried out according to the PN-EN ISO 11358-1:2022 standard [18]. By following the standard, the ramp method was used in the test, in which the sample was tested in a platinum crucible with a constant increase of the furnace temperature by 10 °C per minute to 800 °C. In addition, the airflow was set to 90 dm^3^/min (sample purge) and 10 dm^3^/min nitrogen (balance purge). 

### 2.11. Heat of Combustion 

The heat of combustion of the tested samples was determined using PN-EN ISO 1716:2018 [19] and the digital calorimeter KL-14 (Precyzja-Bit, Bydgoszcz, Poland). The essence of this method is the complete combustion of the sample in an oxygen atmosphere, accurate measurement of the temperature increase, and calculation of the sample’s heat of combustion on this basis. The experiments were performed using refractory vessels (so-called crucibles) made of glass or metal. The sample was placed in a crucible inside a calorimeter bomb filled with oxygen under pressure. The sample was connected to the electrodes of the bomb using an ignition wire. When the ignition was activated, combustion of the sample was induced, and the heat was absorbed by the water surrounding the colorimetric bomb. Accurate measurement of temperature changes was essential. 

### 2.12. Smoke Generation Test

The smoke generation test was performed according to the ISO 5659-2:2017-08 standard, entitled Plastics—Smoke Generation—Part 2: Determination of Optical Density by a Single-Chamber Test [20]. The apparatus for smoke generation tests consisted of a closed chamber, a holder, a radiating cone, a pilot burner, a light transmission and measurement system, and other auxiliary devices to control the working conditions during the test. The test chamber was made of panels made of metal resistant to chemicals and corrosion. The inside dimensions of the chamber were 91.4 × 91.4 (±0.3) × 61 (±0.3) cm. There was a hinged door with a window enabling observation and a removable cover for the window, protecting against light entering the chamber. Inside, there was a blow-out safety plate in the form of aluminum foil with an area of 806 cm^2^. The exhaust panel was protected by a stainless-steel mesh. By following the ISO 5659-2:2017-08 standard, the samples were cut into squares (7.5 × 7.5 cm^2^). Each sample was wrapped along the edges with aluminum foil. The center of the sample (6.5 × 6.5 cm^2^) was exposed to a constant heat irradiance level.

The smoke generated during the experiment is measured by the photometric equipment in the chamber as a function of light transmittance. The following parameters were measured and calculated during the test. 

Transmittance (*T*), where *I* is the light intensity and *I*_0_ is the light intensity without the smoke.

$$T=\frac{I}{I_{0}}$$

Specific optical density (*D_S_*), where *V* is the volume of the chamber [0.501 m^3^], *A* is the sample area exposed to the radiative flux [65 × 65 mm^2^], and *L* is the length of the light beam [0.914 m].

$$D_{S}=\frac{V}{A\times L}\times log\left(\frac{1}{T}\right)$$

*D_S_*_n is the specific optical density measured at n minutes of the experiment, where n = 1–4.

*D_S_*_max_ is the maximum specific optical density.

*VOF*_4_ is the cumulative value of *D_S_* calculated due to the equation:

$$VOF_{4}=D_{S}\_1+D_{S}\_2+D_{S}\_3+\frac{D_{S}\_4}{2}$$

The *VOF*_4_ parameter gives information about the rate of smoke production during the first four minutes of the test.

## 3. Results and Discussion

### 3.1. Pot Life Determination

**Figure 5:**
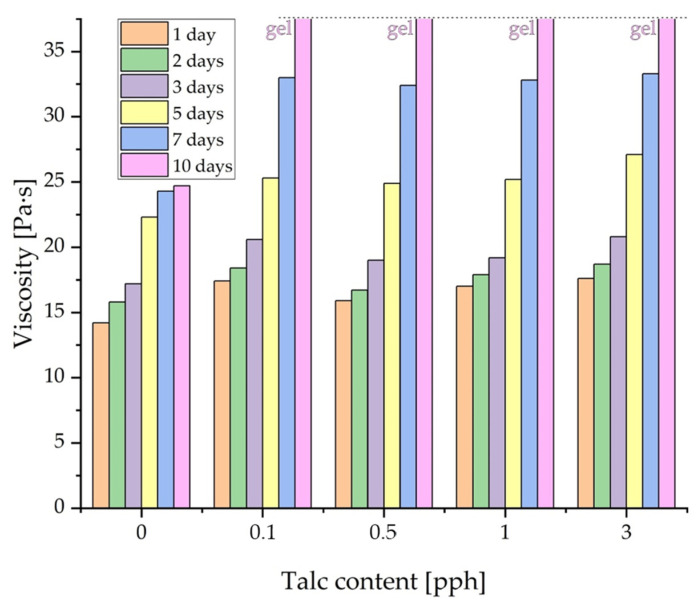
The viscosity of silicone pressure-sensitive adhesives (Q2-7358) with various concentrations of the filler.

Table 2 summarizes the effect of the addition of the highest concentrations of fillers on the viscosity of the adhesive composition over time, so that it was possible to confirm the tendency reported in the literature [21,22] to increase the viscosity over time after the addition of the filler, thanks to which the adhesive composition transforms into an unsusceptible gel form. This problem means that compositions obtained in this way cannot be stored for a long time. In all tested cases, the addition of fillers caused the sample to gel approximately 10 days after its preparation. Changes in the viscosity of compositions with variable talc contents over time are shown in Figure 5.

### 3.2. Application Performance of Pressure-Sensitive Adhesives

**Figure 6:**
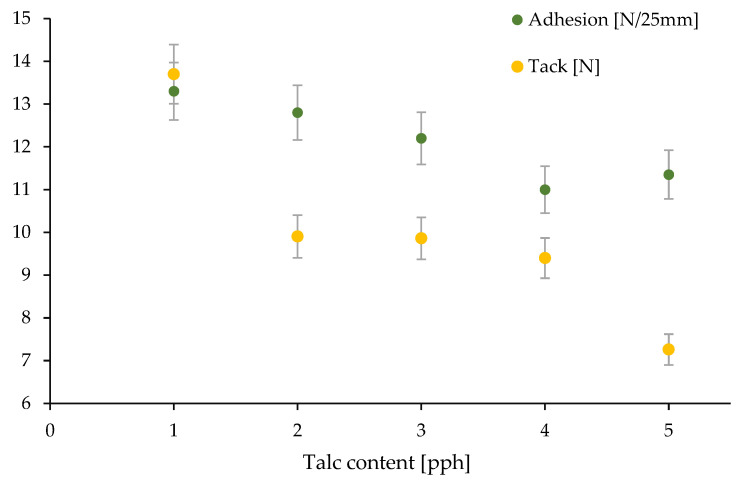
Adhesion and tack test results for silicone pressure-sensitive adhesives with different talc contents.

Figure 6 shows the results of tests on the effects of adding talc to the silicone pressure-sensitive adhesive composition on the adhesion and tack properties. The adhesion and tack values slightly decreased with the talc content. This phenomenon is commonly observed in self-adhesive technologies containing fillers [23,24]. The conventional acceptable limit for adhesion and stickiness, where it is assumed that the tested material can be used as an adhesive tape, is 10 N/25 mm and 8 N, respectively [25]. Only for 3 pph of filler, the samples did not meet the limit values for stickiness.

Table 3 shows the cohesion values at room and elevated temperatures and the SAFT test for silicone adhesive tapes modified with talc. The added filler spread well in the silicone-based matrix. All tested compositions at 23 °C and 70 °C maintained high cohesion values (above 72 h). The results of SAFT tests showed that the presence of talc increased the thermal resistance of adhesives, even at least filling amounts (0.5 and 1.0 pph) of filler. The maximum value was reached (above 225 °C). For comparison, the thermal resistance of the glue without fillers was 150 °C.

Table 4 summarizes the results of the influence of talc addition on the behavior of cross-linked adhesive compositions. Even a small addition of a fine filler resulted in a dramatic decrease in the shrinkage value. Unlike other fillers known from the literature, the effect of increasing shrinkage occurred much faster but did not exceed the value of the composition without the filler. This may be caused by the beginning of agglomeration of the filler evenly distributed in the matrix, as evidenced by the tendency for a uniform increase in shrinkage with increasing filling in the composition [22,26,27,28]. Taking into account these results and the results of adhesion and stickiness tests, it can be concluded that the filler was evenly distributed in the matrix [22,26,28].

### 3.3. DSC Analysis

**Figure 7:**
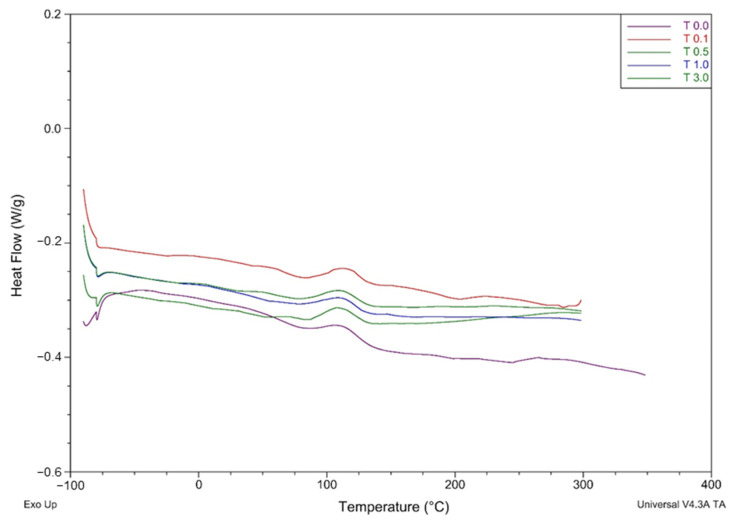
DSC thermograms of developed adhesive compositions.

The thermal effect of the cross-linking process on the obtained adhesive compositions is presented in Table 5 and in Figure 7. With an increase in the amount of talc in the composition, compared to the pure sample, a decrease and then an increase in the values of the thermal effect of the cross-linking reaction were observed. This could be due to the initial good arrangement of polymer chains in the adhesive composition, and then, with the increase in the amount of filler and the appearance of agglomerates (spatial clumps), this effect decreased. With such small additions of fillers, there were no significant changes at the temperature of maximum of exothermal peak assigned to the cross-linking process, and they are rather uneven. Most likely, the addition of such a small amount of filler does not significantly affect the processes occurring during the cross-linking of the resin. A slight increase in T_max_ temperature can be noted for the lowest filler contents, but this is more related to the ordering of polymers in the adhesive composition obtained in this way [22].

### 3.4. Thermogravimetric Analysis (TGA)

**Figure 8:**
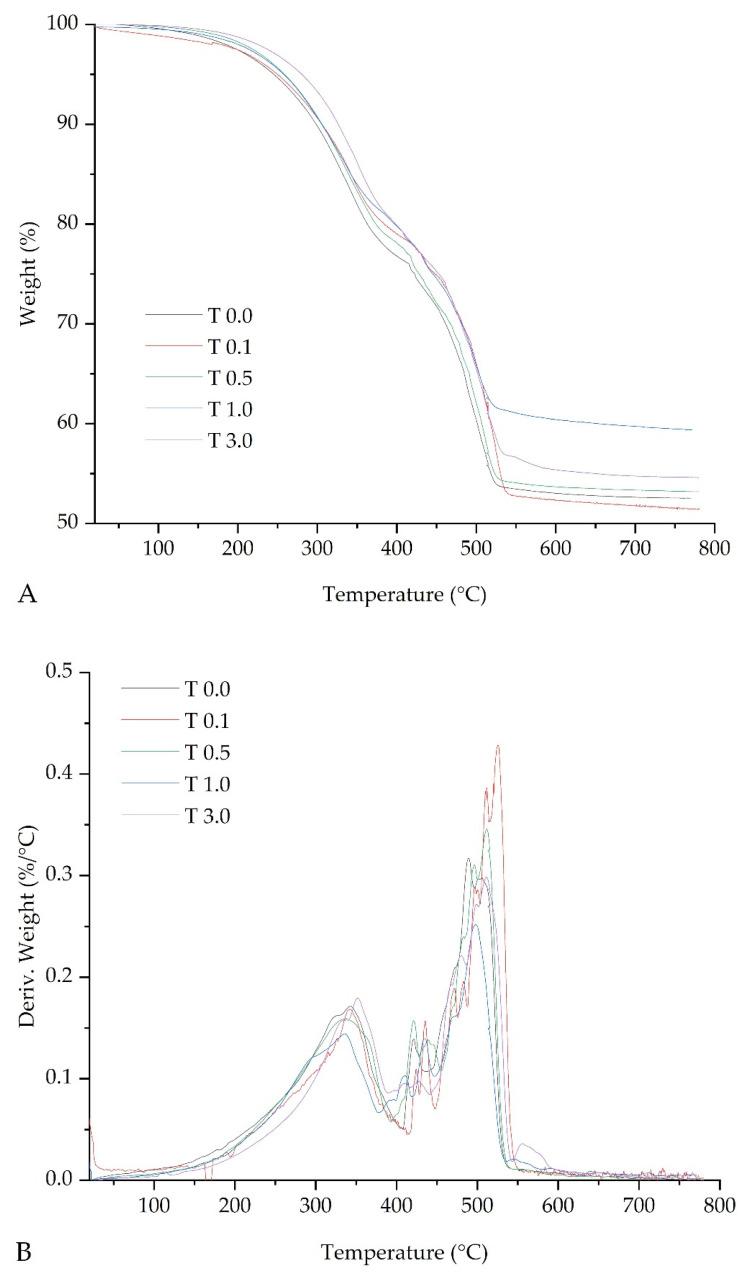
Thermograms of Si-PSA: (**A**) TGA and (**B**) DTG.

The results of the thermogravimetric examination of the adhesive samples are summarized in Figure 8. The mass decrease runs through the two stages in all thermograms. The first stage is assigned to the condensation of the OH groups of the silicone matrix. The second stage is related to the degradation of the polymer matrix [2]. For each sample, more than 50% of the initial mass remained after testing. As the concentration of talc in the tested adhesives increased, the temperature at the beginning of thermal decomposition increased. The highest residue, 54.1%, was found in pure adhesive, while the smallest residue, 51.5%, was found in the adhesive with the addition of 3.0 pph talc. The highest decomposition onset temperature of 281 °C was achieved for the sample of silicone adhesive with the addition of 3.0% talc, while the lowest value of 259 °C was recorded for the pure adhesive. The addition of talc at 1.0 and 3.0 pph also resulted in an increase in the end-of-decomposition temperature. The lowest end-of-decomposition temperature of 532 °C was achieved for sample T 0.5, while the highest end-of-decomposition temperature of 615 °C was achieved by the adhesive with an addition of 1.0% talc. It can be concluded that the results obtained are very similar to the results of other studies described in the literature [29]. 

### 3.5. Heat of Combustion

**Figure 9:**
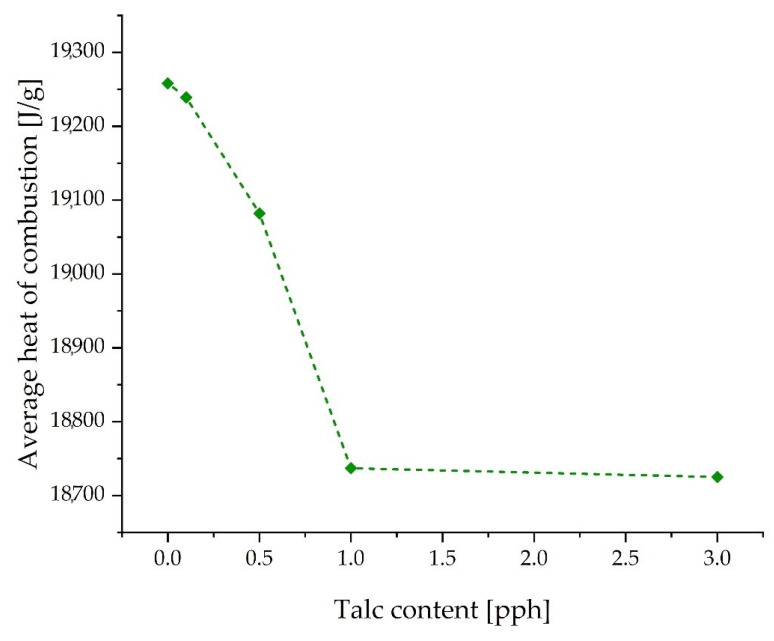
Average heat of combustion of the samples.

The summary results of the heat of combustion tests for individual substances are presented in Table 6 and Figure 9. The obtained heat of combustion results ranged from 18,725 J/g to 19,258 J/g. The decrease in the heat of combustion was directly related to the amount of added filler and decreased with the increase in its content in the composition [30]. The lowest heat of combustion was recorded for the sample T 3.0 (18,725 J/g), while the highest result was achieved by the unmodified glue (19,258 J/g). Modification of the tested adhesives with talc reduces their flammability and the intensity of smoke emissions. This is confirmed by the decrease in the tested heat of combustion with the increase in the addition of talc.

### 3.6. Smoke Generation Test

**Figure 10:**
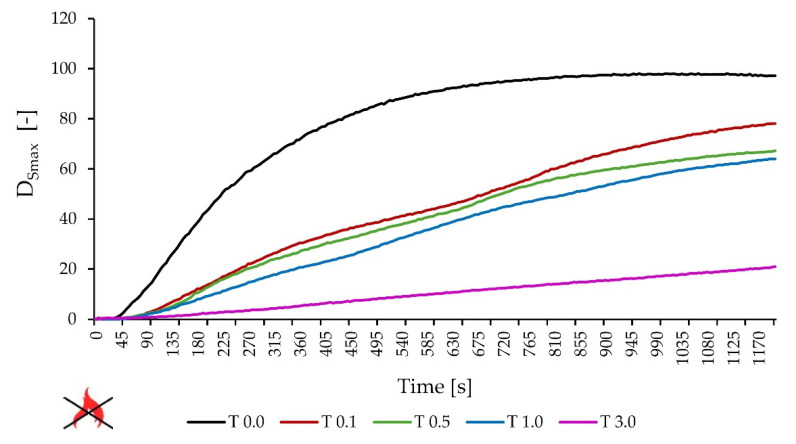
Smoke test—specific optical density vs. time (heat radiation 25 kW/m^2^, without pilot flame).

**Figure 11:**
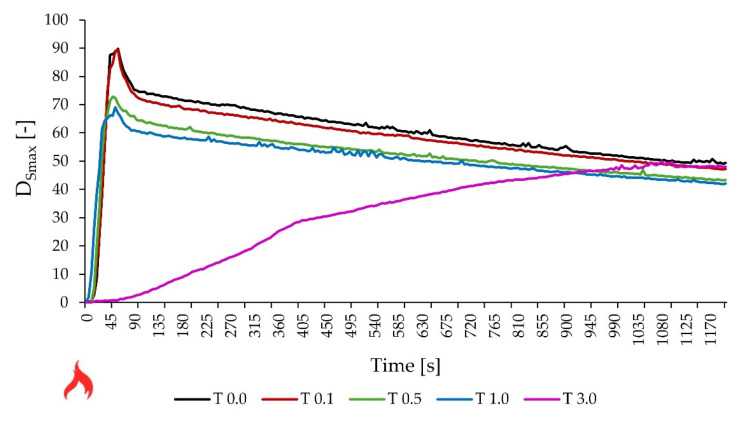
Smoke test—specific optical density vs. time (heat radiation 25 kW/m^2^, with pilot flame).

In Table 7, the results of smoke generation tests carried out at a heat flux of 25 kW/m^2^ without and with a pilot flame were collected. In Figure 10 and Figure 11, the changes in the specific optical density over time (in the heat flux of 25 kW/m^2^) without and with a pilot flame, respectively, are shown. The D_smax_ values achieved were each time significantly higher for a pure adhesive compared to the adhesives with the addition of talc. The lowest D_Smax_ value was recorded in each series of tests for glue with the addition of 3.0 pph of talc and the highest for glue without additives. The ratio of these values was, respectively, 1–4.7 in the case of a heat flux of 25 kW/m^2^ without a pilot flame and 1–1.8 for a heat flux of 25 kW/m^2^ with a pilot flame. Considering the mass loss of the tested samples, it can be concluded that in the case of a lower value of heat flux (25 kW/m^2^) without a external pilot flame, the glue with an admixture of 1.0 pph of talc burned the least, while the second result was achieved by the sample T 3.0 (90% and 70% of the initial weight remained, respectively). With the same flux value but using a pilot flame, the adhesive T 3.0 (50% by weight) remained the most residue. There is a difference between flame and flameless combustion—in the case of combustion without a flame, the time to reach Dsmax is many times longer.

The conducted test results revealed that the addition of talc delayed the thermal decomposition of the adhesive composition and reduced the intensity of smoke emission during combustion as well as the flammability of the entire composition. Thus, talc acted as a fire retardant, thermal stabilizer, and smoke suppressant in the composition [30,31,32].

**Figure 12:**
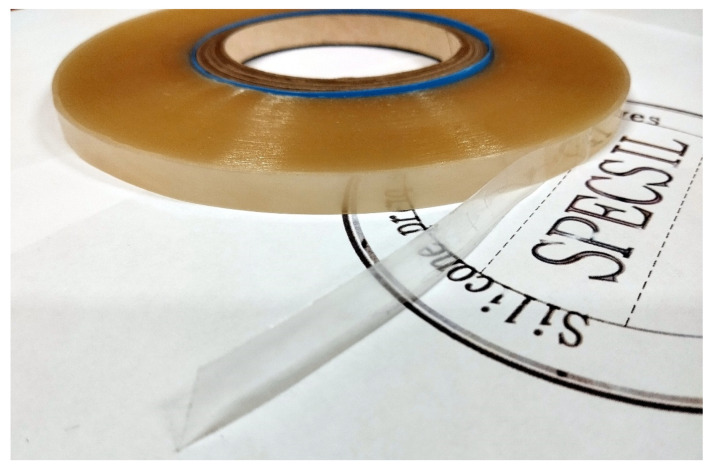
Sample tapes prepared with 0.1 pph of talc on an industrial scale.

Based on the results obtained, one composition was selected for industrial trials. It was a composition containing 0.1 pph of talc as a filler. In cooperation with Taconic Polska Sp. z o. o., a short series of self-adhesive tape (Figure 12) was produced on a fluorosilicone coated PET carrier. The resulting tape was again subjected to the tests described above, and it turned out that it retained all its application properties, as shown in Table 8. The difference in tack and peel adhesion values did not exceed several percent compared to the results of laboratory-scale samples. The slight decrease in these parameters and the improvement in cohesion and SAFT can be explained by a more effective cross-linking process during production on a larger scale. It can therefore be concluded that increasing the production scale for the tested self-adhesive tape was successful. 

## 4. Conclusions

Novel talc-modified silicone pressure-sensitive adhesives have been successfully obtained. The carried-out tests of the composite materials confirmed the maintenance of high cohesion values and improvement of heat-resistant properties and shrinkage, while, at the same time, slightly (within the standards) decreasing the adhesion and stickiness values of the modified tapes compared to the tape without filler in the composition. 

Moreover, the conducted flammability tests showed that the talc used can act as a natural additive, improving the fire-retardant properties of silicone adhesives. It can be assumed that its admixture does not increase the toxicity of the thermal decomposition products and combustion of such adhesives because talc itself decomposes at high temperatures without releasing harmful substances [14].

Additional tests carried out on an industrial scale allowed for the production of self-adhesive tapes with talc on an industrial scale. The tests confirmed that the properties achieved on a laboratory scale were maintained.

The obtained compositions, and therefore self-adhesive products based on them, such as self-adhesive tapes, can be used in the automotive industry, for example. 

## Figures and Tables

**Table 1:** Symbols used for Si-PSA systems with talc.

Filler	Filler Content (pph)	Symbols
Talc	0.0	T 0.0
	0.1	T 0.1
	0.5	T 0.5
	1.0	T 1.0
	3.0	T 3.0

**Table 2:** The viscosity of silicone pressure-sensitive adhesives (Q2-7358) with various concentrations of the filler.

Sample	Viscosity (Pa·s)					
	1 Day	2 Days	3 Days	5 Days	7 Days	10 Days
T 0.0	14.2	15.8	17.2	22.3	24.3	24.7
T 0.1	17.4	18.4	20.6	25.3	33.0	Gel
T 0.5	15.9	16.7	19.0	24.9	32.4	Gel
T 1.0	17.0	17.9	19.2	25.2	32.8	Gel
T 3.0	17.6	18.7	20.8	27.1	33.3	Gel

**Table 3:** Cohesion and SAFT test results for silicone pressure-sensitive adhesives with different talc contents.

Sample	Cohesion (h)		SAFT (°C)
	23 °C	70 °C	
T 0.0	>72	>72	150
T 0.1	>72	>72	>225
T 0.5	>72	>72	>225
T 1.0	>72	>72	>225
T 3.0	>72	>72	>225

**Table 4:** Shrinkage of silicone pressure-sensitive adhesives with different talc contents.

Sample	Shrinkage (%)											
	10 min	30 min	1 h	3 h	8 h	24 h	2 Days	3 Days	4 Days	5 Days	6 Days	7 Days
T 0.0	0.412	0.421	0.641	0.900	1.020	1.140	1.325	1.325	1.325	1.325	1.325	1.325
T 0.1	0.047	0.064	0.069	0.075	0.139	0.181	0.185	0.207	0.217	0.323	0.451	0.507
T 0.5	0.080	0.184	0.189	0.195	0.259	0.301	0.305	0.327	0.337	0.373	0.531	0.557
T 1.0	0.119	0.182	0.250	0.286	0.342	0.407	0.409	0.427	0.438	0.468	0.568	0.596
T 3.0	0.169	0.332	0.440	0.496	0.542	0.607	0.609	0.627	0.648	0.683	0.743	0.743

**Table 5:** The effect of talc concentration on the maximum temperature and thermal effect of the cross-linking process of the adhesive films.

Sample	T_max_ (°C) of Exothermal Peak	ΔH (J/g)
T 0.0	111	4.1
T 0.1	113	3.8
T 0.5	110	3.9
T 1.0	108	4.2
T 3.0	112	4.2

**Table 6:** Residual mass of Si-PSA samples with various filler contents.

Acronym	Sample Mass Before Test (g)	Mass of the Wire (g)	Mass of Residue after Burning (g)
T 0.0	1.0000	0.0061	0.8154
T 0.1	1.0001	0.0060	0.8428
T 0.5	1.0000	0.0059	0.8376
T 1.0	1.0000	0.0060	0.8196
T 3.0	1.0001	0.0060	0.8278

**Table 7:** Smoke properties of Si-PSA samples.

Adhesives Sample	T 0.0		T 0.1		T 0.5		T 1.0		T 3.0	
Pilot Flame	Without	With	Without	With	Without	With	Without	With	Without	With
Initial weight, m_p_ (g)	1.0	1.0	1.0	1.0	1.0	1.0	1.0	1.0	1.0	1.0
End weight, m_k_ (g)	0.1	0.1	0.3	0.1	0.5	0.1	0.9	0.1	0.7	0.5
D_smax_ (–)	98.01	89.78	78.10	89.43	64.28	72.91	64.05	69.07	20.98	49.91
Time to D_smax_ (s)	1065	60	1200	60	1200	50	1200	55	1200	1080
D_s__4 (–)	53.36	70.06	18.43	67.19	17.45	59.95	12.40	57.65	2.92	13.56
VOF_4 (–)	90.41	263.9	26.38	256.25	23.36	229.86	17.48	224.10	5.08	20.66
Burn time (s)	-	50	-	60	-	49	-	44t	-	-

**Table 8:** Test results for industrial-scale manufactured tape T 0.1 Ind compared with the properties of laboratory obtained sample (T 0.1).

Sample	Adhesion (N/25mm)	Tack (N)	Cohesion (h)		SAFT (°C)
			23 °C	70 °C	
T 0.1	12.8	9.9	>72	>72	>225
T 0.1 Ind.	12,4	9.6	>72	>72	>225

## Data Availability

Data are contained within the article.

## References

[1] Lin S.B., Durfee L.D., Ekeland R.A., McVie J., Schalau G.K. (2007). Recent advances in silicone pressure-sensitive adhesives. J. Adhes. Sci. Technol..

[2] Benedek I., Feldstein M.M. (2008). Technology of Pressure-Sensitive Adhesives and Products.

[3] Mecham S., Sentman A., Sambasivam M. (2010). Amphiphilic silicone copolymers for pressure sensitive adhesive applications. J. Appl. Polym. Sci..

[4] Fitzgerald D.M., Colson Y.L., Grinstaff M.W. (2023). Synthetic pressure sensitive adhesives for biomedical applications. Prog. Polym. Sci..

[5] Lee B.K., Ryu J.H., Baek I., Kim Y., Jang W.I., Kim S., Yoon Y.S., Kim S.H., Hong S., Byun S. (2017). Silicone-Based Adhesives with Highly Tunable Adhesion Force for Skin-Contact Applications. Adv. Healthc. Mater..

[6] II G.K.S., Bobenrieth A., Huber R.O., Nartker L.S., Thomas X. (2018). Silicone Adhesives in Medical Applications. Applied Adhesive Bonding in Science and Technology.

[7] He M., Zhang Q.Y., Guo J.Y. (2011). Synthesis and Characterization of Silicone Based Pressure Sensitive Adhesive. Adv. Mater. Res..

[8] Zhang J., Luo R., Jiang M., Xiang Q., Li J. (2011). The preparation and performance of a novel room-temperature-cured heat-resistant adhesive for ceramic bonding. Mater. Sci. Eng. A.

[9] Rothon R. (2017). Talcs. Fillers for Polymer Applications.

[10] (1998). The use of special talcs as supplementary flame retardants. Addit. Polym..

[11] Morgan A.B., Gilman J.W. (2013). An overview of flame retardancy of polymeric materials: Application, technology, and future directions. Fire Mater..

[12] Yan L., Tang X., Xu Z., Xie X. (2022). Fabrication of talc reinforced transparent fire-retardant coating towards excellent fire protection, antibacterial, mechanical and anti-ageing properties. Polym. Degrad. Stab..

[13] Ewell R.H., Bunting E.N., Geller R.F. (1935). Thermal decomposition of talc. J. Res. Natl. Bur. Stand..

[14] Liu X., Liu X., Hu Y. (2014). Investigation of the thermal decomposition of talc. Clays Clay Miner..

[15] Ulian G., Valdrè G. (2015). Density functional investigation of the thermophysical and thermochemical properties of talc [Mg3Si4O10(OH)2]. Phys. Chem. Miner..

[16] Sanghvi M.R., Tambare O.H., More A.P. (2022). Performance of various fillers in adhesives applications: A review. Polym. Bull..

[17] FINAT (2019). Technical Handbook Test Methods.

[18] (2022). Plastics—Thermogravimetry (TG) of Polymers—Part 1: General Principles.

[19] (2018). Reaction to Fire Tests for Products—Determination of the Gross Heat of Combustion (Calorific Value).

[20] (2017). Plastics—Smoke Generation—Part 2: Determination of Optical Density by a Single-Chamber Test.

[21] Szadkowski B., Marzec A., Rybiński P., Żukowski W., Zaborski M. (2020). Characterization of Ethylene–propylene Composites Filled with Perlite and Vermiculite Minerals: Mechanical, Barrier, and Flammability Properties. Materials.

[22] Antosik A.K., Makuch E., Gziut K. (2022). Influence of modified attapulgite on silicone pressure-sensitive adhesives properties. J. Polym. Res..

[23] Pramanik S., Karak N. (2017). Polymer Nanocomposites for Adhesive, Coating, and Paint Applications. Properties and Applications of Polymer Nanocomposites.

[24] Antosik A.K., Mozelewska K. (2022). Influence of Nanoclay on the Thermo-Mechanical Properties of Silicone Pressure-Sensitive Adhesives. Materials.

[25] Weisbrodt M., Kowalczyk A. (2022). Self-Crosslinkable Pressure-Sensitive Adhesives from Silicone-(Meth)acrylate Telomer Syrups. Materials.

[26] Leong Y.W., Abu Bakar M.B., Ishak Z.A.M., Ariffin A., Pukanszky B. (2004). Comparison of the mechanical properties and interfacial interactions between talc, kaolin, and calcium carbonate filled polypropylene composites. J. Appl. Polym. Sci..

[27] Zhang X., He J., Yue L., Bai Y., Liu H. (2019). Heat resistance of acrylic pressure-sensitive adhesives based on commercial curing agents and UV/heat curing systems. J. Appl. Polym. Sci..

[28] Joo H.-S., Do H.-S., Park Y.-J., Kim H.-J. (2006). Adhesion performance of UV-cured semi-IPN structure acrylic pressure sensitive adhesives. J. Adhes. Sci. Technol..

[29] Yadav R., Singh M., Shekhawat D., Lee S.-Y., Park S.-J. (2023). The role of fillers to enhance the mechanical, thermal, and wear characteristics of polymer composite materials: A review. Compos. Part A Appl. Sci. Manuf..

[30] Hamdani S., Longuet C., Perrin D., Lopez-cuesta J.-M., Ganachaud F. (2009). Flame retardancy of silicone-based materials. Polym. Degrad. Stab..

[31] Singh B., Sharma N. (2008). Mechanistic implications of plastic degradation. Polym. Degrad. Stab..

[32] Wang Z., Huang Z., Yang T. (2020). Silica coated expanded polystyrene/cement composites with improved fire resistance, smoke suppression and mechanical strength. Mater. Chem. Phys..

